# Efficacy of the Mendelsohn maneuver combined with repetitive facilitation principles in poststroke dysphagia: A randomized controlled trial based on videofluoroscopic swallow study

**DOI:** 10.1097/MD.0000000000050466

**Published:** 2026-08-28

**Authors:** Wenjing Jia, Caihong Cui, Liang Qin, Jialin Sun, Chunxiao Wan

**Affiliations:** a Department of Rehabilitation Medicine, General Hospital of Tianjin Medical University, Tianjin, China; b Department of Rehabilitation Medicine, Affiliated Hospital of Hebei University, Baoding, Hebei, China.

**Keywords:** dysphagia, Mendelsohn maneuver, repetitive facilitation principles, stroke, videofluoroscopic swallow study

## Abstract

**Background:**

This study aimed to evaluate the effects of Mendelsohn maneuver training combined with repetitive facilitation principles on swallowing function in patients with poststroke dysphagia.

**Methods:**

This prospective, assessor-blinded randomized controlled trial assessed 60 patients for eligibility and randomly assigned 56 eligible participants to a control or experimental group. The control group received conventional indirect swallowing therapy, whereas the experimental group received the Mendelsohn maneuver combined with repetitive facilitation principles. Treatment duration and therapist contact were matched between groups. Training was delivered for 20 minutes per session, 5 times weekly, for 4 weeks. Outcomes included the Penetration–Aspiration Scale, pharyngeal residue, upper esophageal sphincter opening diameter, Dysphagia Outcome and Severity Scale, and nasogastric tube removal.

**Results:**

All 56 randomized participants completed treatment and posttreatment assessment. Both groups showed improvement in swallowing function. Compared with the control group, the experimental group had lower Penetration–Aspiration Scale scores at Day 28 and greater improvement in Dysphagia Outcome and Severity Scale scores. Findings for pharyngeal residue were inconsistent and should be considered exploratory: vallecular residue change favored the experimental group, whereas posttreatment scores did not differ, and no significant difference was found in pyriform sinus residue change. No significant group differences were observed in upper esophageal sphincter opening improvement or nasogastric tube removal.

**Conclusion:**

The combined intervention may provide additional benefits for swallowing safety and overall swallowing function in patients with poststroke dysphagia.

## 1. Introduction

Dysphagia is a clinical syndrome characterized by difficulty or abnormality during swallowing and may be classified as oropharyngeal dysphagia and esophageal dysphagia, of which oropharyngeal dysphagia primarily involves the oral and pharyngeal phases.^[1]^ Previous systematic reviews have shown a high prevalence of dysphagia in patients with acute stroke, although reported estimates vary according to the timing of assessment and the screening methods used.^[2]^ Dysphagia may present with a range of clinical manifestations. Problems may arise in the oral phase, such as impaired mastication and reduced ability to form, manipulate, or transport the bolus; or in the pharyngeal phase, such as reduced hyoid movement, pharyngeal residue, and aspiration/laryngeal penetration.^[3]^ Dysphagia may lead to complications such as malnutrition, dehydration, or aspiration pneumonia and, in severe cases, may even result in death.^[4]^

Behavioral interventions for dysphagia generally include compensatory strategies and rehabilitative exercises. Compensatory strategies are intended to improve swallowing safety and efficiency immediately, whereas rehabilitative exercises use active, repetitive task practice to improve swallowing function.^[5]^ The Mendelsohn maneuver requires the patient to voluntarily prolong peak hyolaryngeal elevation during swallowing and may be used either as a compensatory maneuver or as a rehabilitative exercise.^[6]^ Previous studies have shown that it can prolong hyoid and laryngeal elevation and improve coordination between hyolaryngeal movement and upper oesophageal sphincter (UES) opening, although its effect on maximum UES opening diameter remains uncertain.^[7]^

Successful performance of the Mendelsohn maneuver depends on the patient’s ability to initiate laryngeal elevation, recognize peak elevation, and sustain that position.^[7–9]^ After stroke, impaired sensorimotor control may make it difficult to initiate or maintain laryngeal elevation and may lead to compensatory effort, resulting in inconsistent performance.^[9–11]^ Repetitive facilitation exercise, also known as the Kawahira method, combines facilitation with high-repetition practice. It pairs voluntary motor intention with sensory and manual cues matched to the intended movement, with the aim of eliciting the target action and reinforcing a more accurate movement pattern through repetition.^[12]^

In the present study, the principles of repetitive facilitation exercise were incorporated into Mendelsohn training. The Mendelsohn maneuver provided a clear, task-specific goal: active initiation of hyolaryngeal elevation followed by maintenance at its peak. Tactile stimulation of the suprahyoid muscles, manual assistance in the direction of laryngeal elevation, and synchronized verbal cues were used to help patients initiate and sustain the target movement. Combining these approaches may increase the likelihood of successful task completion and improve the consistency of repeated practice, thereby providing more effective task-specific training and potentially improving hyolaryngeal motor control and swallowing safety. The present study therefore used videofluoroscopic swallowing study to evaluate the effects of this combined intervention in patients with poststroke dysphagia.

Accordingly, the present study evaluated Mendelsohn maneuver training combined with repetitive facilitation principles in patients with poststroke dysphagia. Videofluoroscopic swallow study (VFSS) was used to assess physiological swallowing outcomes, including penetration–aspiration, pharyngeal residue, and maximum UES opening diameter. Clinical outcomes, including Dysphagia Outcome and Severity Scale (DOSS) scores and nasogastric tube removal, were also assessed.

## 2. Methods

### 2.1. Study design

This was a prospective, assessor-blinded, 2-arm, parallel-group, randomized controlled superiority trial with a 1:1 allocation ratio. The trial was conducted in the Department of Rehabilitation Medicine, Affiliated Hospital of Hebei University, Baoding, China, from April 2025 to April 2026. No important changes were made to the trial protocol after trial commencement.

### 2.2. Participants

Inclusion criteria were as follows: age 20 to 80 years; imaging-confirmed cerebral hemorrhage or cerebral infarction (including brainstem lesions), with time since onset < 6 months; dysphagia confirmed by VFSS, accompanied by liquid penetration or aspiration; presence of a basic swallowing reflex and ability to cooperate with swallowing assessment; ability to use at least 1 upper limb to cooperate with rehabilitation training; and no obvious cognitive impairment [mini-mental state examination score > 22].

Exclusion criteria were as follows: cerebellar stroke; dysphagia that predated the index stroke, including persistent dysphagia following a previous cerebral infarction or intracerebral hemorrhage, including a previous brainstem stroke, or dysphagia caused by other conditions such as traumatic brain injury, head and neck tumors, myasthenia gravis, Parkinson disease, or motor neuron disease; unstable vital signs or severe underlying diseases, including serious dysfunction of vital organs such as the heart, liver, lungs, or kidneys, or malignant tumors; and coma, severe cognitive impairment, severe aphasia, or communication difficulties that prevented cooperation with assessment and treatment.

The study was approved by the institutional ethics committee of the Affiliated Hospital of Hebei University (approval No. HDFYLL-HT-2025-074), and all participants provided written informed consent. The trial was retrospectively registered with the Chinese Clinical Trial Registry (registration No. ChiCTR2600120665; registration date: March 18, 2026; URL: https://www.chictr.org.cn/showproj.html?proj=307650).

### 2.3. Patient and public involvement

Patients and the public were not involved in the design, conduct, reporting, or dissemination plans of this trial.

### 2.4. Randomization

Eligible patients were randomly assigned to the experimental or control group in a 1:1 ratio using a random number table generated by an independent researcher. Simple randomization was used without stratification or blocking. No formal allocation concealment mechanism was used. Treating therapists and patients had no access to the allocation sequence before assignment. Because of the nature of the rehabilitation intervention, participants and therapists could not be blinded after allocation. VFSS assessors were blinded to group allocation.

### 2.5. Interventions

#### 2.5.1. Swallowing management provided to both groups

All participants received standard stroke care and comprehensive rehabilitation. Throughout the study, nutritional support, diet texture, liquid consistency, feeding position, feeding rate, bolus volume, oral care, and nasogastric tube management were adjusted by the clinical team according to clinical swallowing assessment and videofluoroscopic swallowing study findings. The same clinical principles were applied to both groups.

Participants considered safe for oral intake received food and liquids of an appropriate texture and consistency, together with measures such as upright or semi-upright positioning, smaller bolus volumes, a slower feeding rate, and longer intervals between boluses. Participants who were not considered safe for oral intake continued to receive nasogastric tube feeding. Nutritional route and oral intake recommendations were adjusted according to changes in swallowing function, independently of group allocation.

Both study interventions were delivered as indirect swallowing therapy. No food or liquid was used as a training bolus during the treatment sessions, and routine meals and clinical feeding management were not included in the 20-minute intervention period. During the study, participants were not permitted to receive additional dysphagia-specific treatments outside the study protocol, including neuromuscular electrical stimulation, pharyngeal electrical stimulation, acupuncture, respiratory muscle training, transcranial magnetic stimulation, transcranial direct-current stimulation, or additional swallowing maneuvers. All training was performed within the participant’s tolerance and was modified or discontinued if it caused pain, pronounced gagging, persistent coughing, or other discomfort.

#### 2.5.2. Control group

In addition to the swallowing management described above, the control group received standardized conventional indirect swallowing therapy consisting of orofacial exercises and thermal-tactile stimulation.^[13]^ Orofacial exercises included lip closure, lip protrusion, cheek puffing, tongue protrusion and retraction, lateral tongue movements, and tongue elevation. Verbal cues and movement guidance were provided according to each participant’s ability, with brief rest periods allowed when necessary.

Thermal-tactile stimulation was applied to the bilateral faucial pillars and posterior pharyngeal wall using a chilled cotton swab. The frequency and interval of stimulation were adjusted according to individual tolerance. Treatment was delivered individually by a therapist experienced in dysphagia rehabilitation for 20 minutes per session, 5 sessions per week, for 4 consecutive weeks.

#### 2.5.3. Experimental group

In addition to common swallowing management, the experimental group received Mendelsohn maneuver training combined with repetitive facilitation principles instead of the conventional indirect swallowing therapy provided to the control group.

Training was performed using saliva swallows. The target movement consisted of active initiation of swallowing, upward movement of the hyolaryngeal complex, and maintenance of peak laryngeal elevation for approximately 5 seconds, followed by relaxation.^[14]^ Participants were seated upright with the head and neck in a neutral position.

Before each swallow, the therapist lightly tapped the suprahyoid region between the hyoid bone and mandible while giving the verbal cue, “Get ready and lift your larynx upward.” When the participant initiated swallowing and the larynx began to elevate, the therapist gently supported the thyroid cartilage in the direction of laryngeal elevation. At peak elevation, the participant was instructed to “Hold it; do not relax” and to maintain the position for approximately 5 seconds. Light tapping over the suprahyoid region continued during the holding phase. The therapist then instructed the participant to relax, withdrew the tactile and manual input, and allowed the larynx to return naturally to its resting position before the next repetition.

The therapist monitored for compensatory movements, including neck extension, excessive chin elevation, shoulder elevation, and excessive generalized effort. When these occurred, the participant’s position, the amount of manual assistance, and the verbal cues were adjusted to facilitate a more appropriate movement pattern.

Five target swallows were performed per set, with brief rest periods between sets according to fatigue and tolerance. Each session included at least 50 target swallows. Treatment was delivered individually for 20 minutes per session, 5 sessions per week, for 4 consecutive weeks, by therapists experienced in dysphagia rehabilitation who had received standardized training in the intervention protocol.

### 2.6. Outcome measures

#### 2.6.1. VFSS assessment

Swallowing function was assessed using VFSS. Images were acquired at 30 frames/second, and the swallowing process was recorded synchronously. The contrast agent was prepared by mixing iohexol and water at a ratio of 1:1. According to the grading criteria of the International Dysphagia Diet Standardisation Initiative,^[15]^ a xanthan gum-based thickener was used to prepare test materials of different viscosities. In this study, an International Dysphagia Diet Standardisation Initiative level 2 mildly thick liquid was mainly used as the assessment stimulus, with a dose of approximately 5 mL administered each time by the caregiver. Participants swallowed voluntarily when ready, and no additional prompts were given during the assessment in order to simulate the natural swallowing state as far as possible.^[16]^ All VFSS images were rated independently by 2 assessors experienced in swallowing assessment; if discrepancies arose, the final result was determined through discussion between the 2 assessors.

#### 2.6.2. Penetration–Aspiration Scale (PAS): primary outcome

The PAS was proposed by Rosenbek et al^[17]^ in 1996. It is an 8-point scale used to assess the degree to which material enters the airway and whether it is expelled from the airway. Scoring is based on the position of the contrast material in the airway and its clearance. A score of 1 indicates no penetration or aspiration; scores of 2 to 5 indicate penetration; and scores of 6 to 8 indicate aspiration. Higher scores indicate poorer swallowing safety.

#### 2.6.3. Opening diameter of the UES

UES opening was measured on VFSS. The image frame showing the greatest UES opening during swallowing was selected, captured using frame-by-frame analysis software, and measured with ImageJ software. A metal ball marker placed behind the ear (diameter 6 mm) was used for calibration, and the maximum UES opening diameter was calculated.

#### 2.6.4. Pharyngeal residue score

Pharyngeal residue included vallecular residue and pyriform sinus residue. With reference to the scoring method proposed by Eisenhuber et al,^[18]^ retained contrast material on swallowing images was graded as follows: 0, no residue; 1, mild residue (< 25% of structural height); 2, moderate residue (25–50%); and 3, severe residue (> 50%).

#### 2.6.5. DOSS

The DOSS was developed by O’Neil et al^[19]^ in 1999. This 7-level rating system is used to grade the severity of swallowing dysfunction according to swallowing safety, diet level, and the patient’s independence in eating. The scale has demonstrated good reliability and validity in clinical swallowing assessment.^[20]^

#### 2.6.6. Nasogastric tube removal assessment

Nasogastric tube removal was assessed after 4 weeks only in participants who had a tube in place at baseline. The same criteria were applied in both groups. Tube removal was considered when clinical swallowing assessment and videofluoroscopic swallowing study findings indicated that oral intake was safe with the prescribed food texture and liquid consistency, with no aspiration observed (PAS score < 6); oral intake met at least 75% of the estimated daily energy and fluid requirements for 3 consecutive days without reliance on tube feeding for primary nutrition or hydration; the prescribed oral feeding plan was tolerated for 3 consecutive days without recurrent coughing, persistent wet voice, marked dyspnea, clinically significant oxygen desaturation, or meal-limiting fatigue; respiratory and general medical status remained stable, with no aspiration pneumonia or feeding-related respiratory deterioration; and the participant or primary caregiver was able to follow the recommended feeding precautions. The decision was made jointly by a rehabilitation physician and a swallowing therapist who were not involved in delivering the interventions, with any disagreement resolved through discussion with the clinical team. Nasogastric tube removal rate was defined as the proportion of participants with a tube at baseline who met these criteria and underwent successful removal after treatment. Only participants with a nasogastric tube at baseline were included in the analysis, and removal rates were compared using the chi-square test or Fisher exact test when expected cell counts were below 5.

In this study, the PAS score was the primary outcome, whereas the DOSS score, pharyngeal residue, maximum UES opening diameter, and tube removal rate were secondary outcomes. Outcomes were assessed at baseline and after 4 weeks of treatment.

#### 2.6.7. Harms

Adverse events were monitored throughout the intervention period, including aspiration pneumonia, choking episodes requiring medical intervention, fatigue, discomfort, dizziness, or any other treatment-related events.

## 3. Statistical analysis

### 3.1. Sample size calculation

The primary outcome of this study was the PAS score. The PAS is an ordinal scale ranging from 1 to 8. For the purpose of sample size estimation, it was treated as an approximately continuous variable, consistent with previous randomized controlled trials in poststroke dysphagia. Sample size calculation was performed with reference to the method reported by Alyanak et al.^[21]^ A standardized effect size of 0.80 was adopted to provide a conservative estimate. Using PASS software (version 23.0.2; NCSS, LLC, Kaysville), with a 2-sided *α* of 0.05, statistical power of 80%, and a 1:1 allocation ratio, the required sample size was calculated as 26 participants per group.Allowing for an anticipated dropout rate of approximately 10%, 30 participants were planned for each group (total n = 60). A total of 56 participants completed the study, with 28 in each group, meeting the minimum sample size requirement. No interim analyses were planned or performed, and no formal stopping guidelines were specified.

### 3.2. Data analysis

Data were analyzed using SPSS 27.0 (IBM Corp., Armonk). Measurement data conforming to a normal distribution are presented as mean ± standard deviation; between-group comparisons were performed using the independent-samples *t*-test, and within-group pre- and posttreatment comparisons were performed using the paired *t*-test. Non-normally distributed data and ordinal data are presented as median (quartiles); between-group comparisons were performed using the Mann–Whitney *U* test, and within-group comparisons were performed using the Wilcoxon signed-rank test. Count data are presented as number of cases and percentage, and between-group comparisons were performed using the *χ*^2^ test or Fisher exact test. The PAS was the primary outcome, and the DOSS, pharyngeal residue, maximum UES opening diameter, and tube removal rate were secondary outcomes. All tests were 2-sided, and *P* < .05 was considered statistically significant. Analyses were conducted using participants who completed the intervention and had available posttreatment outcome data. No imputation was performed for missing outcome data, and analyses were conducted using available cases. No ancillary, subgroup, or sensitivity analyses were prespecified or performed. Secondary outcome analyses were not adjusted for multiplicity.

## 4. Results

### 4.1. Baseline characteristics

**Figure 1.**
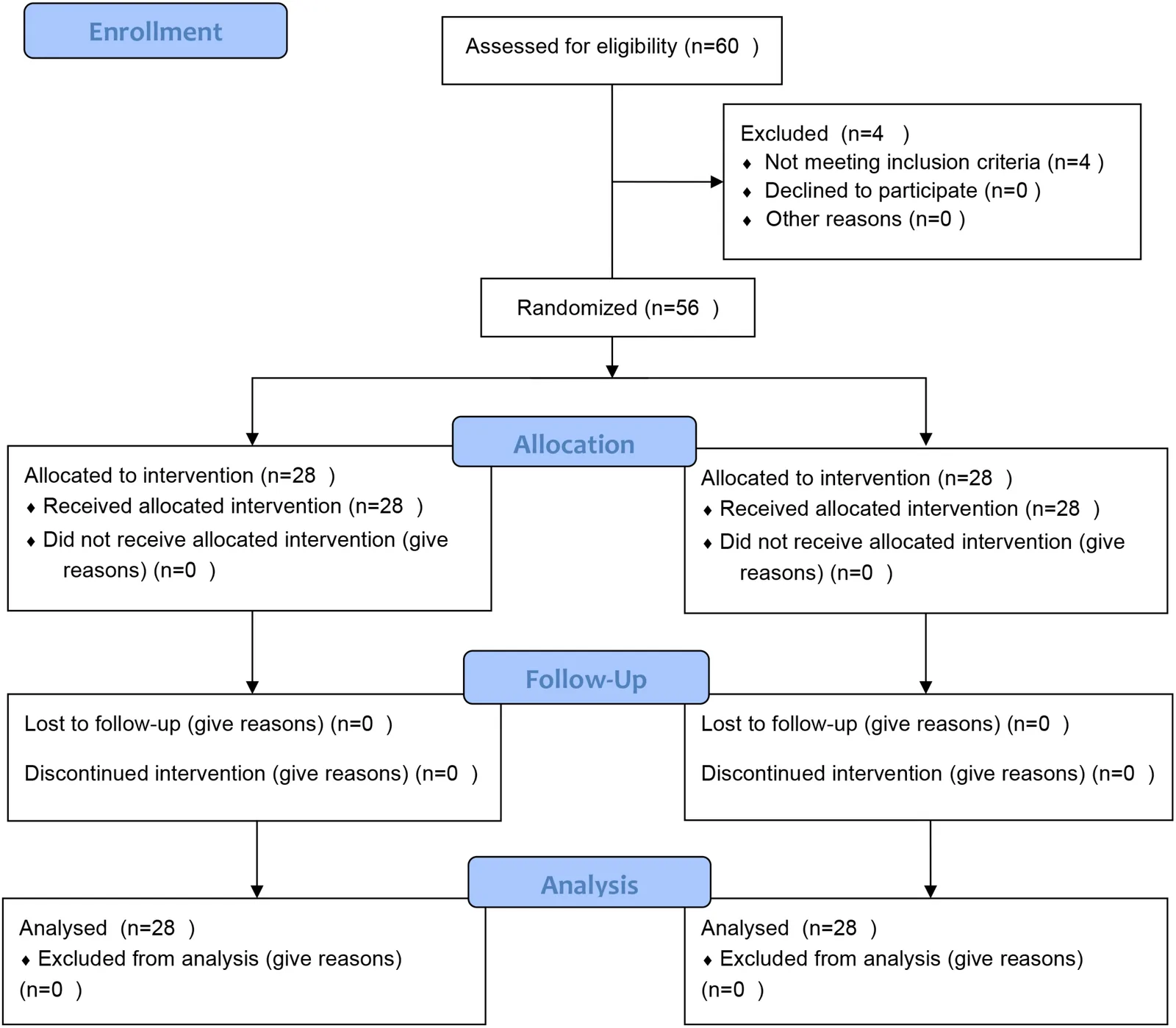
CONSORT flow diagram. CONSORT =Consolidated Standards of Reporting Trials, n = number of participants.

A total of 60 patients were assessed for eligibility. Four patients were excluded before randomization because they were found not to meet the eligibility criteria. Finally, 56 eligible patients were randomly assigned to the control group or the experimental group, with 28 patients in each group (Fig. 1). All randomized participants received the allocated intervention, completed the 4-week intervention and posttreatment VFSS assessment, and were included in the final analysis. The mean age of the participants was 60.64 ± 10.76 years, and 37 (66.1%) were male. The median time since onset was 30 days. Patients with brainstem lesions accounted for 46.4% of the sample. There were no statistically significant differences between the 2 groups in age, sex, stroke type, lesion site, days since onset, or baseline PAS score (*P* > .05), indicating comparability between the groups (Table 1). All participants included in the final analysis completed the 4-week intervention and posttreatment VFSS assessment. The assigned interventions were delivered as planned by experienced therapists. The trial ended as planned after completion of recruitment and follow-up.

**Table 1:** Comparison of baseline characteristics between the 2 groups.

	Overall (n = 56)	Control group (n = 28)	Experimental group (n = 28)	*P*
Age	60.64 ± 10.76	61.29 ± 12.16	60.00 ± 9.32	.659*
Sex, n (%)				1.000***
Male	37 (66.1)	18 (64.3)	19 (67.9)	
Female	19 (33.9)	10 (35.7)	9 (32.1)	
Stroke type, n (%)				.528***
Cerebral infarction	43 (76.8)	20 (71.4)	23 (82.1)	
Cerebral hemorrhage	13 (23.2)	8 (28.6)	5 (17.9)	
Lesion site, n (%)				1.000***
Basal ganglia	30 (53.6)	15 (53.6)	15 (53.6)	
Brainstem	26 (46.4)	13 (46.4)	13 (46.4)	
Days since onset (IQR)	30 (16.5/50.5)	32 (18/53.5)	23.5 (15/46)	.218**
PAS-T0	7.0 (7.0, 7.0)	7.0 (7.0, 7.0)	7.0 (7.0, 7.0)	.130**

IQR = interquartile range, n = number of participants, PAS-T0 = Penetration–Aspiration Scale before treatment.

* Independent-samples *t*-test.

** Mann–Whitney *U* test.

*** Chi-squared test.

### 4.2. PAS results: primary outcome

#### 4.2.1. Statistical analysis of PAS scores

PAS scores were treated as ordinal data and are presented as the median and first and third quartiles (M [Q1, Q3]). Because the intervals between adjacent PAS categories cannot be assumed to be equal and baseline scores were highly clustered, PAS score distributions at Day 28 were compared directly between groups using the Mann–Whitney *U* test. Within-group changes from baseline to Day 28 were assessed using the Wilcoxon signed-rank test. Change in PAS score was defined as dPAS = baseline PAS − Day 28 PAS, with a positive value indicating improved swallowing safety. Between-group differences in dPAS were examined as a supportive analysis using the Mann–Whitney *U* test. Effect size was calculated as *Zr* = |*Z*|/√N, where N was the total number of participants included in the relevant comparison. All tests were 2-sided, and *P* < .05 was considered statistically significant.

#### 4.2.2. PAS results

At baseline, PAS scores were concentrated at 7 in both groups, with a median of 7.0 (7.0, 7.0) in both the control and experimental groups. By Day 28, PAS scores had decreased in both groups. The median score was 7.0 (2.5, 7.0) in the control group and 2.0 (2.0, 5.0) in the experimental group. Direct comparison at Day 28 showed that the distribution of PAS scores was lower in the experimental group than in the control group (Mann–Whitney *U* = 205.000, *Z* = −3.158, *P* = .002, *r* = 0.42) (Table 2), indicating better swallowing safety after treatment in the experimental group. Within-group analyses showed a reduction in PAS scores from baseline in both groups (control group: *Z* = −3.184, *P* = .001; experimental group: *Z* = −3.910, *P* < .001).

**Table 2:** Comparison of PAS scores between the 2 groups.

	Control group median (Q1, Q3)	Experimental group median (Q1, Q3)	* *P*
Before treatment	7.0 (7.0, 7.0)	7.0 (7.0, 7.0)	
After treatment	7.0 (2.5, 7.0)	2.0 (2.0, 5.0)	.002
Change	0.5 (0.0, 3.5)	4.5 (2.0, 5.0)	.003
** *P*	.001	< .001	

Q1–Q3: interquartile range.

PAS = Penetration–Aspiration Scale.

* *P* value for between-group analysis (Mann–Whitney *U* test).

** *P* value for within-group analysis (Wilcoxon signed-rank test).

In the supportive analysis, the median dPAS was 0.5 (0.0, 3.5) in the control group and 4.5 (2.0, 5.0) in the experimental group. The distribution of improvement was greater in the experimental group than in the control group (Mann–Whitney *U* test, *P* = .003, *r* = 0.39). As the PAS is an ordinal scale, these findings were interpreted in terms of differences in score distributions and ranks rather than as a fixed physiological change measured on an equal-interval scale.

### 4.3. DOSS results

DOSS data were available for all 28 participants in each group at baseline and after treatment. DOSS scores were analyzed as ordinal data and are presented as median (Q1, Q3). Within-group comparisons between baseline and posttreatment were performed using the Wilcoxon signed-rank test. Between-group comparisons of improvement (ΔDOSS = DOSS at day 28 − DOSS at baseline) were conducted using the Mann–Whitney *U* test. A 2-sided significance level of 0.05 was used. At baseline, there was no significant difference in DOSS scores between the experimental and control groups. Within-group analyses showed significant improvement in both groups after treatment. Improvement in DOSS score was greater in the experimental group than in the control group (median ΔDOSS 2.5 [1.5, 3.0] vs 1.0 [1.0, 2.0]) (Table 3). The Hodges–Lehmann median difference was 1.0 point (95% confidence interval [CI] 0.0–2.0). The between-group difference was statistically significant (Mann–Whitney *U* test, *P* = .002, *r* = 0.41), indicating a moderate treatment effect.

**Table 3:** Comparison of DOSS scores between the 2 groups.

	Control group median (Q1, Q3)	Experimental group median (Q1, Q3)	* *P*
Before treatment	3 (2, 3)	3 (2, 4)	.799
After treatment	4 (3, 5)	5 (5, 6)	.002
ΔDOSS	1 (1, 2)	2.5 (1.5, 3)	.002
** *P*	< .001	< .001	

Q1–Q3: interquartile range.

DOSS = Dysphagia Outcome and Severity Scale.

* *P* value for between-group analysis (Mann–Whitney *U* test).

** *P* value for within-group analysis (Wilcoxon signed-rank test).

### 4.4. Pharyngeal residue grading

Pharyngeal residue was rated on a 4-point ordinal scale ranging from 0 to 3. Baseline scores were generally low and clustered between 0 and 1, leaving limited room for improvement. Vallecular residue decreased after treatment in both groups. The median change was 0.0 (0.0, 1.0) in the control group and 1.0 (0.0, 2.0) in the experimental group, with a nominally significant difference in the unadjusted between-group comparison (Mann–Whitney *U* test, *P* = .032). However, the groups did not differ significantly in their Day 28 scores (*P* = .120), and the Hodges–Lehmann estimate of the location shift was 0 (95% CI, 0–1), suggesting that the between-group effect was small (Table 4).

**Table 4:** Comparison of vallecular residue scores between the 2 groups.

	Control group median (Q1, Q3)	Experimental group median (Q1, Q3)	** *P*
Pretreatment vallecular residue	1.0 (0.0, 1.0)	1.0 (0.0, 1.5)	.631
Posttreatment vallecular residue	0.5 (0.0, 1.0)	0.0 (0.0, 1)	.120
Change (Δ)	0.0 (0.0, 1.0)	1.0 (0.0, 2.0)	.032
* *P*	.008	< .001	

* *P* value for within-group analysis (Wilcoxon signed-rank test).

** *P* value for between-group analysis (Mann–Whitney *U* test).

Pyriform sinus residue also decreased after treatment in both groups. Although the Day 28 scores differed between groups (*P* = .012) (Table 5), the between-group comparison of change scores was not significant [control group, 0.0 (0.0, 1.0); experimental group, 0.5 (0.0, 1.0); *P* = .215]. The Hodges–Lehmann estimate of the location shift was 0 (95% CI, 0–1). These findings do not provide consistent evidence that the combined intervention produced additional improvement in pyriform sinus residue.

**Table 5:** Comparison of pyriform sinus residue scores between the 2 groups.

	Control group median (Q1, Q3)	Experimental group median (Q1, Q3)	** *P*
Pretreatment pyriform sinus residue	1.0 (1.0, 1.5)	1.0 (1.0, 1.0)	.339
Posttreatment pyriform sinus residue	1.0 (1.0, 1.0)	0.5 (0.0, 1.0)	.012
Change (Δ)	0.0 (0.0, 1.0)	0.5 (0.0, 1.0)	.215
* *P*	.002	< .001	

* *P* value for within-group analysis (Wilcoxon signed-rank test).

** *P* value for between-group analysis (Mann–Whitney *U* test).

**Figure 2.**
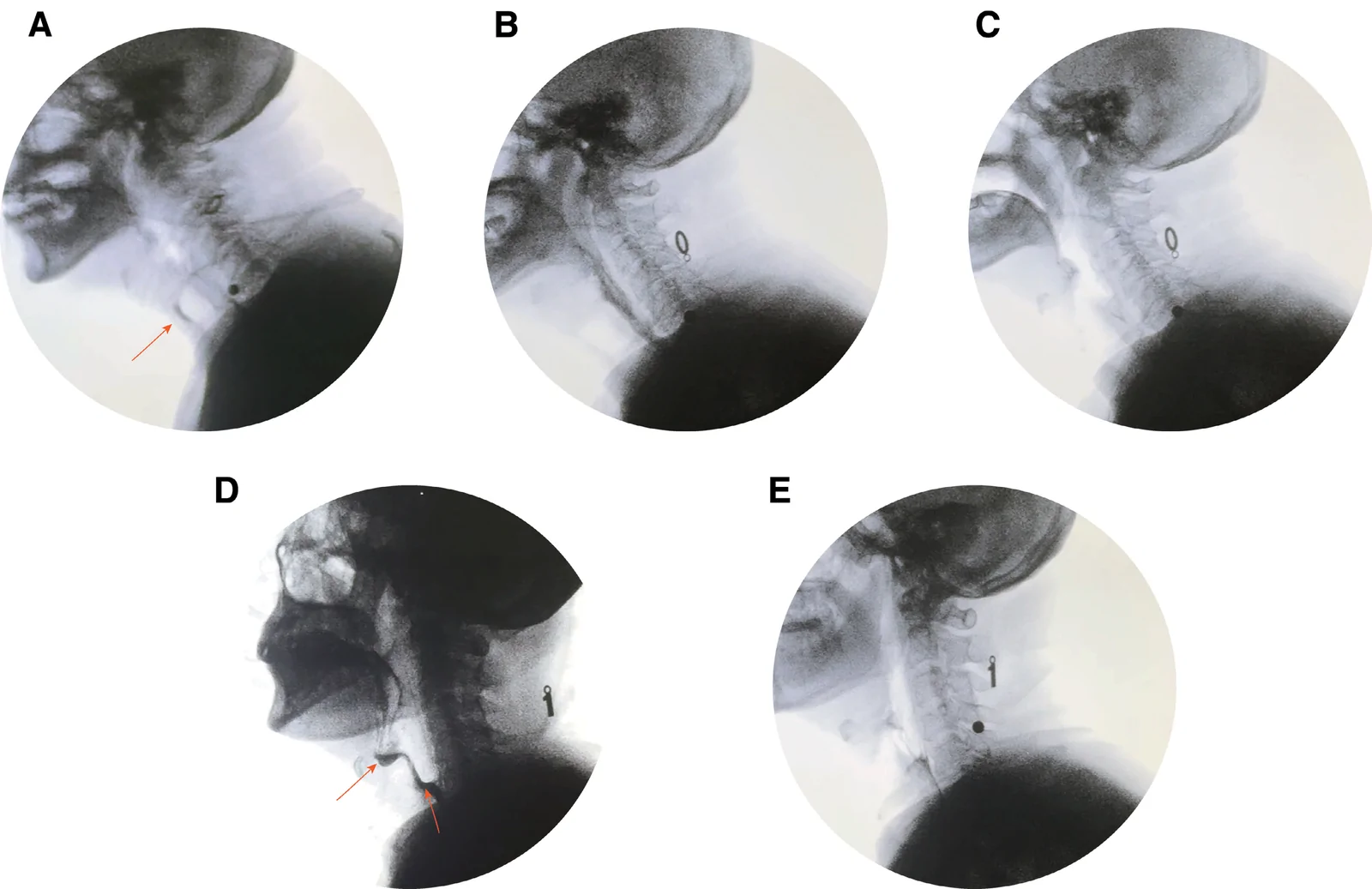
Representative videofluoroscopic images. Panels (A–C) are from the same case and show improvement in aspiration. (A) Aspiration before treatment (PAS = 7); (B) no penetration or aspiration during swallowing after treatment; and (C) no penetration or aspiration after swallowing following treatment. Panels (D–E) are from the same case and show improvement in pharyngeal residue. (D) Vallecular residue (*) and pyriform sinus residue (#) were visible after swallowing before treatment; (E) after treatment, no vallecular residue was observed after swallowing, and only a small amount of pyriform sinus residue (#) remained.

Overall, the findings were not consistent across the different analyses. The change in vallecular residue favored the experimental group, but this was not supported by the Day 28 comparison or the estimated location shift. For pyriform sinus residue, the Day 28 difference was not accompanied by a significant between-group difference in change scores. These findings, therefore, do not establish a consistent additional benefit of the combined intervention on pharyngeal residue and should be regarded as exploratory. Representative VFSS images illustrating changes in aspiration and pharyngeal residue before and after treatment are shown in Figure 2.

### 4.5. Opening diameter of the UES

The baseline and Day 28 UES opening diameters were approximately normally distributed and are presented as mean ± standard deviation. Between-group comparisons at each time point were performed using independent-samples *tt*-tests. Because the change scores [dUES = UES at Day 28 − UES at baseline] were not normally distributed, they are presented as median (Q1, Q3) and were compared between groups using the Mann–Whitney *U* test. Within-group changes from baseline to Day 28 were assessed using the Wilcoxon signed-rank test. All tests were 2-sided, with a significance level of *α* = 0.05.

Before treatment, the maximum UES opening diameters were 5.043 ± 1.636 mm in the control group and 5.000 ± 1.725 mm in the experimental group, with no statistically significant difference between the 2 groups (*t* = 0.095, *P* = .924). After treatment, the maximum UES opening diameters were 5.664 ± 1.359 mm in the control group and 5.950 ± 1.546 mm in the experimental group, again with no statistically significant difference between the 2 groups (*t* = −0.734, *P* = .466).

Within-group comparisons showed that UES opening diameter increased significantly from baseline to Day 28 in both groups (control group: *Z* = −4.404, *P* < .001; experimental group: *Z* = −4.546, *P* < .001). The median change was 0.50 mm (0.20, 0.85) in the control group and 0.80 mm (0.30, 1.10) in the experimental group. However, the between-group difference in change scores was not statistically significant (Mann–Whitney *U* = 284.000, *Z* = −1.776, *P* = .076). Complete baseline and posttreatment UES opening diameter data were available for all 28 participants in each group (Table 6).

**Table 6:** Comparison of UES opening diameter between the 2 groups.

	Control group mean (mm)	Experimental group mean (mm)	* *P*
Before treatment	5.043 ± 1.636	5.000 ± 1.725	.924
After treatment	5.664 ± 1.359	5.950 ± 1.546	.466
Change, median (Q1, Q3)	0.50 (0.20, 0.85)	0.80 (0.30, 1.10)	.076
** *P*	< .001	< .001	

* *P* value for between-group analysis (independent-samples *t*-test).

** *P* value for within-group analysis (Wilcoxon signed-rank test).

### 4.6. Tube removal rate

The tube removal outcome was binary data. The tube removal rate was defined as the proportion of patients with a nasogastric tube before treatment who successfully underwent tube removal after treatment. Between-group comparison was performed using the chi-squared test; when the expected frequency was < 5, Fisher exact test was used. The significance level was *α* = 0.05, and all tests were 2-sided. Among the patients who had a nasogastric tube in place before treatment, 24 were in the control group, of whom 17 successfully had the tube removed, giving a tube removal rate of 70.8%; 27 were in the experimental group, of whom 23 successfully had the tube removed, giving a tube removal rate of 85.2%. There was no statistically significant difference in tube removal rate between the 2 groups (*χ*^2^ = 1.547, *P* = .214). The tube removal rate was higher in the experimental group than in the control group, but the difference did not reach statistical significance. The absolute risk difference was 14.4 percentage points, and the relative risk was 1.20 in favor of the experimental group (Table 7).

**Table 7:** Comparison of tube removal rates between the 2 groups.

Group	Tube not removed, n (%)	Successful tube removal, n (%)	Total	*P*
Control group	7 (29.2)	17 (70.8)	24	
Experimental group	4 (14.8)	23 (85.2)	27	
				.214

N = number of participants.

### 4.7. Harms

No intervention-related adverse events or unintended events were observed in either group during the study period.

## 5. Discussion

This study found that both conventional indirect swallowing therapy and Mendelsohn maneuver training combined with repetitive facilitation principles improved swallowing function in patients with poststroke dysphagia. At the end of treatment, the distribution of PAS scores was lower in the experimental group, and improvement in DOSS scores was also greater. These findings suggest that the combined intervention may offer additional benefits for swallowing safety and overall swallowing function. However, consistent between-group differences were not found for pharyngeal residue, maximum UES opening diameter, or nasogastric tube removal. The findings therefore support a possible benefit for selected swallowing outcomes rather than a general advantage across all measures. As the PAS is an ordinal scale, the results should be interpreted in terms of score distributions and ranks rather than as fixed changes on an equal-interval scale.

Poststroke dysphagia is closely related to impaired sensorimotor control of swallowing following injury to the central nervous system.^[22]^ Swallowing is a complex sensorimotor process involving the cerebral cortex, subcortical structures, and brainstem swallowing centers.^[23,24]^ Successful swallowing depends not only on sufficient muscle force but also on sensory input, movement initiation, timing, and coordination between muscle groups. After stroke, patients may have difficulty initiating laryngeal elevation, maintaining the elevated position, or coordinating the swallowing sequence. Some compensate by extending the neck, lifting the chin excessively, or increasing general effort. Although greater effort may help some patients complete a swallow, it does not necessarily produce an accurate or stable target movement and may recruit muscles that are not directly involved in the task.^[25]^ Similar compensatory effort and abnormal synergies are commonly observed in other motor tasks after stroke. Swallowing rehabilitation may therefore need to address not only muscle function but also the initiation, maintenance, and repeated performance of the target movement.

The Mendelsohn maneuver has long been used in dysphagia rehabilitation.^[14]^ It requires the patient to voluntarily prolong peak hyolaryngeal elevation during swallowing and is intended to train both the duration of laryngeal elevation and its coordination with UES opening. Previous studies have shown that the maneuver can prolong hyoid and laryngeal elevation and may also increase the duration of UES opening.^[9]^ Intensive Mendelsohn training has also been associated with changes in the duration of hyoid movement and UES opening in patients with poststroke dysphagia. In the present study, patients were asked to maintain peak laryngeal elevation for approximately 5 seconds, allowing repeated practice of both elevation and sustained holding.

The intervention also incorporated selected principles of repetitive facilitation exercise. This approach emphasizes voluntary movement intention, sensory and manual input matched to the direction of the target movement, and repeated practice.^[26]^ Previous work on repetitive facilitation exercise has mainly focused on limb rehabilitation after stroke, where it has been associated with improvements in motor function. The limb-based protocol was not transferred directly to swallowing in the present study.^[27,28]^ Instead, active movement intention, tactile stimulation, movement-congruent manual assistance, and high-repetition practice were adapted to the task of laryngeal elevation. The Mendelsohn maneuver provided a clear movement goal, while the facilitation techniques may have helped patients who found it difficult to initiate or maintain elevation independently. The potential value of combining these approaches may lie less in encouraging greater effort than in increasing the number of successful and accurately performed repetitions. Further work using physiological measures is needed to clarify the mechanisms underlying this response.

The pharyngeal residue findings require cautious interpretation. Residue in the valleculae and pyriform sinuses was rated using a relatively coarse ordinal scale, and baseline scores were generally low. Many participants had either no residue or only mild residue, leaving limited room for further improvement. Although the between-group comparison of change in vallecular residue reached nominal statistical significance, the groups did not differ significantly in their Day 28 scores, and the Hodges–Lehmann estimate of the location shift was 0. In addition, no adjustment for multiple secondary outcome comparisons was applied. Therefore, the observed difference was small, was not consistent across analyses, and should be regarded as exploratory rather than as firm evidence of an additional treatment effect. Change in pyriform sinus residue did not differ significantly between groups.

Maximum UES opening diameter increased after treatment in both groups, but the degree of change did not differ significantly between groups. The Mendelsohn maneuver may have a more direct effect on the duration of hyolaryngeal elevation than on maximum opening diameter. Measuring diameter alone may therefore provide an incomplete account of UES function. Differences in lesion location may also have influenced the response to treatment. Nearly half of the participants had brainstem lesions, which may involve the swallowing centers and related neural pathways and make recovery of swallowing timing and UES control more complex. As no analysis stratified by lesion location was performed, this explanation remains speculative.

From a clinical perspective, the Mendelsohn maneuver combined with repetitive facilitation principles manual training may provide a practical form of indirect swallowing therapy for patients with poststroke dysphagia. The intervention combines a clearly defined laryngeal elevation task with tactile cueing, movement-congruent manual assistance, and repeated practice. It may be particularly useful for patients who have difficulty performing the Mendelsohn maneuver independently. The present findings support a possible benefit for swallowing safety and overall swallowing function, although no clear additional advantage was demonstrated for pharyngeal residue, maximum UES opening diameter, or nasogastric tube removal.

Several limitations should be considered. The sample size was relatively small and may have limited the ability to detect between-group differences in some secondary outcomes. Follow-up was restricted to the treatment period, so the durability of the observed effects remains unknown. Participants were not stratified according to lesion location, time since stroke, or dysphagia severity, which limited further interpretation of treatment response. Pharyngeal residue was evaluated using a 4-category scale, and the low baseline scores may have produced a floor effect. The analysis also included several secondary outcomes, increasing the possibility of chance findings. Both interventions were delivered as indirect swallowing therapy without food or liquid used as training material, and the results are therefore most applicable to similar treatment protocols. Formal allocation concealment was not used, and the nature of the intervention prevented blinding of participants and therapists, introducing possible selection and performance bias. Finally, the proposed effects on neural control and motor coordination were inferred from the intervention and clinical outcomes rather than measured using electromyography, neuroimaging, or other neurophysiological methods.^[29]^ Future studies should include larger samples, longer follow-up, stratification by lesion characteristics, and more sensitive measures of swallowing timing and residue. Direct neurophysiological assessment may also help clarify the mechanisms underlying the combined intervention.

## Acknowledgments

The authors thank all participants and clinical staff involved in this study.

## Author contributions

**Conceptualization:** Wenjing Jia, Caihong Cui, Chunxiao Wan.

**Data curation:** Wenjing Jia, Caihong Cui.

**Formal analysis:** Wenjing Jia, Caihong Cui, Jialin Sun.

**Funding acquisition:** Chunxiao Wan.

**Investigation:** Caihong Cui, Liang Qin, Jialin Sun.

**Methodology:** Wenjing Jia, Liang Qin.

**Project administration:** Liang Qin, Chunxiao Wan.

**Resources:** Chunxiao Wan.

**Supervision:** Chunxiao Wan

**Writing – original draft:** Wenjing Jia.

**Writing – review & editing:** Caihong Cui, Liang Qin, Jialin Sun, Chunxiao Wan.
